# Physicians’ preferencesfor radioiodine treatment of differentiated thyroid cancer in Brazil: an observational study

**DOI:** 10.20945/2359-4292-2023-0228

**Published:** 2024-06-19

**Authors:** Rosália do Prado Padovani, Isabella Fagian Pansani, Marília Martins Silveira Marone, Fernanda Vaisman, Ana Luiza Silva Maia, José Miguel Silva Dora, Helton Estrela Ramos, Ana Amélia Fialho de Oliveira Hoff, George Barbério Coura

**Affiliations:** 1 Faculdade de Ciências Médicas Santa Casa de Misericórdia de São Paulo São Paulo SP Brasil Faculdade de Ciências Médicas, Santa Casa de Misericórdia de São Paulo, São Paulo, SP, Brasil; 2 Instituto Nacional do Câncer Rio de Janeiro RJ Brasil Instituto Nacional do Câncer, Rio de Janeiro, RJ, Brasil; 3 Universidade Federal do Rio Grande do Sul Porto Alegre RS Brasil Universidade Federal do Rio Grande do Sul, Porto Alegre, RS, Brasil; 4 Universidade Federal da Bahia Salvador BA Brasil Universidade Federal da Bahia, Salvador, BA, Brasil; 5 Instituto do Câncer do Estado de São Paulo São Paulo SP Brasil Instituto do Câncer do Estado de São Paulo (Icesp), São Paulo, SP, Brasil

**Keywords:** Thyroid nodule, thyroid neoplasms, radiopharmaceuticals, thyroid cancer, papillary thyroid carcinoma, thyrotropin, thyroid diseases

## Abstract

**Objective:**

The aim of this observational, cross-sectional study was to investigate physicians’ preferences for radioiodine (RAI) treatment in patients with differentiated thyroid cancer (DTC) in Brazil and the factors influencing RAI indications.

**Materials and methods:**

A survey was distributed to physicians potentially involved in DTC care in Brazil to understand the factors influencing RAI indications. The survey collected information on the profiles of the physicians, along with the characteristics of their workplaces and their preferences regarding RAI indications in three hypothetical clinical cases. Cases 1, 2, and 3 described the cases of patients with DTC and variations to the case that included different scenarios to assess how the respondents would change their RAI recommendations. The analysis included the RAI indications across different medical specialties.

**Results:**

A total of 175 physicians answered the survey. There was considerable variability in RAI recommendations in all three cases. The training background influenced the respondents' preferences for RAI indications and their approaches to preparing patients for RAI treatment.

**Conclusion:**

The findings of this study reaffirm the need for a Brazilian consensus among physicians across multiple specialties to help guide health care professionals treating patients with DTC in Brazil.

## INTRODUCTION

The traditional management of differentiated thyroid cancer (DTC) typically includes total thyroidectomy, radioiodine (RAI) treatment, and levothyroxine suppressive therapy. This approach is commonly described as a “one-size-fits-all” strategy. The origin of this approach dates back to the 1970s when most patients with DTC were diagnosed with advanced disease. Based on results from their own retrospective studies, Mazzaferri and cols. (1), Samaan and cols. (2), and Seidlin and cols. (3) recommended a more aggressive management of patients with DTC. Notably, RAI has been used for the treatment of thyroid disease since 1946 (3).

Given the increasing incidence of DTC in recent decades – primarily due to the detection of low-risk tumors – and the risks and potential side effects of RAI treatment, the therapeutic goals and outcomes of DTC have been revised to avoid unnecessary treatments, particularly in patients categorized as low risk or intermediate risk according to the American Thyroid Association (ATA) guidelines (4).

In recent years, important changes have occurred in the indications for RAI treatment and the preparation of patients for this therapy. Categorizing the patient according to staging system is an important step in the selection process for treatment (4). Once patients are categorized within the staging system, the objective of RAI treatment – whether for ablation, adjuvant therapy, or treatment of metastatic disease – is evaluated; this evaluation is key to deciding the therapeutic activity to prescribe (5). This decision is assisted by critical factors considered in the therapeutic decision, including postoperative thyroglobulin levels and imaging tests (cervical ultrasound, diagnostic whole-body scintigraphy with or without single-photon emission computed tomography [SPECT], and SPECT with computed tomography [SPECT/CT]) (6).

The adequate preparation for RAI involves a serum thyroid-stimulating hormone (TSH) level > 30 mIU/L and a low-iodine diet (4,7). Traditionally, achieving this TSH level required withdrawing thyroid hormone replacement. However, the development of recombinant human TSH (rhTSH; Thyrogen, Genzyme, Cambridge, MA, USA) now allows for the achievement of elevated TSH levels without hypothyroidism (8). Initial studies included as potential candidates for rhTSH only those patients categorized as low risk according to the ATA guidelines (8). However, recent studies have shown that rhTSH can also be used in patients with intermediate-risk or high-risk DTC without evidence of distant metastases (9-11). Therefore, rhTSH has become a safe and effective alternative to thyroid hormone withdrawal (7).

As the management of patients with DTC continues to evolve, the adoption of this updated management approach varies considerably worldwide (5). Studies have shown substantial variations in RAI treatment for patients with thyroid cancer and differences in treatment preferences within regions of the same country (12-14).

Based on these considerations, the aim of this study was to uncover the rationale behind RAI prescriptions for DTC in Brazil and the factors that influence physicians’ indications for RAI treatment and dose. Information on the specific preparation used for RAI (*i.e.*, low-iodine diet and thyroid hormone withdrawal *versus* rhTSH) was also obtained.

## MATERIALS AND METHODS

This observational, cross-sectional study was conducted in accordance with the rules of the Research Ethics Committee and was approved in *Plataforma Brasil* under the Certificate of Presentation for Ethical Appreciation (CAAE) number 44107621.3.0000.0068.

A survey (Supplementary Material) was distributed to physicians potentially involved in thyroid cancer care in Brazil to understand the factors considered in RAI indications, recommended RAI activity, and preparations for RAI (*i.e.*, low-iodine diet, thyroid hormone withdrawal *versus* rhTSH). The survey was distributed by email through medical societies that supported the present study, including the Latin American Thyroid Society (LATS), the Brazilian Society of Head and Neck Surgery (SBCC), the Brazilian Society of Endocrinology and Metabolism (SBEM), and the Brazilian Society of Nuclear Medicine (SBMN). The survey was also distributed across WhatsApp groups of physicians from various specialties.

The first part of the survey gathered general information on the respondents regarding age, specialty (endocrinology, general medicine, head and neck surgery, general surgery, nuclear medicine, oncology, or other), their geographic location in Brazil, whether they live in a capital or non-capital city, size of the city they live in terms of number of inhabitants, whether they work in the public or private sector or both, and the average number of patients with thyroid carcinoma they see a month. The second part of the survey gathered information about the profile of the physician’s workplace and particularities about their general practice of recommending RAI, including questions about routinely requesting whole-body scanning (WBS) before RAI; the availability of rhTSH and the situations where they use it; the adoption of a dosimetry protocol and, if so, which protocol (full-body dosimetry, lesion dosimetry, or both) and in which situations they applied dosimetry (renal insufficiency, pediatrics, suspicion of metastasis, pulmonary metastasis, or following multidisciplinary discussions); and the criteria used for RAI dosing (whether following the guidelines by the ATA, European Thyroid Association [ETA], Society of Nuclear Medicine and Molecular Imaging [SNMMI], European Association of Nuclear Medicine [EANM], or other/none). The third part of the survey collected information on the physicians’ recommendation of RAI in three hypothetical clinical cases (Table 1).

**Table 1:** Description of the three hypothetical clinical cases presented to the survey participants

Case 1	A 45-year-old female patient with a 2.0 cm papillary thyroid carcinoma with a follicular variant histology without any other features.
Case 2	A patient with a follicular carcinoma measuring 3.5 cm with invasion of two or five blood vessels.
Case 3	A patient with a tall-cell variant of papillary thyroid carcinoma with two metastatic level VI lymph nodes (1.2 and 0.6 cm) who underwent radioiodine treatment with an activity of 100 mCi in the previous year and has a rising serum thyroglobulin of 109 ng/mL. The patient has no evidence of disease on cervical ultrasound and chest computed tomography shows scattered micronodules.

The analysis of the collected data considered the RAI practices across different Brazilian regions, medical specialties, and health care systems (public and private).

### Statistical analysis

Categorical data are reported as absolute numbers and percentages. Continuous data with normal distribution are reported as mean ± standard deviation, while those with non-normal distribution are reported as median and interquartile range (IQR). The Student’s *t* test or Mann-Whitney test was used to compare mean or median values depending on the normality of the distribution and variance of the continuous data.

The statistical analyses were performed using the software Statistical Package for the Social Sciences (SPSS), Version 25.0 (IBM Corp, Armonk, NY, USA). All the analyses were performed with two-tailed tests, and p values < 0.05 were considered statistically significant.

## RESULTS

**Figure 1:**
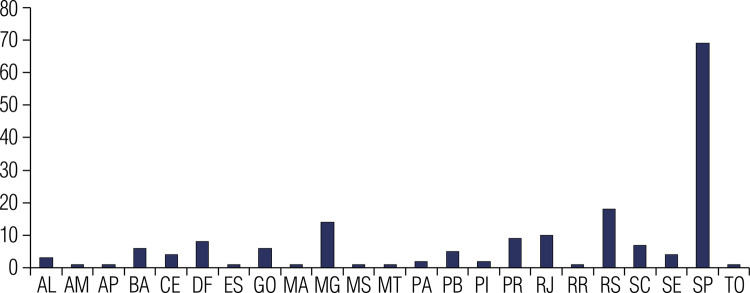
Geographic distribution of survey respondents. The graph illustrates the proportional distribution of physicians who participated in the survey across Brazilian states.

Abbreviations: AL, Alagoas; AM, Amazonas; AP, Amapá; BA, Bahia; CE, Ceará; DF, Distrito Federal; ES, Espírito Santo; GO, Goiás; MA, Maranhão; MG, Minas Gerais; MS, Mato Grosso do Sul; MT, Mato Grosso; PA, Pará; PB, Paraíba; PI, Piauí; PR, Paraná; RJ, Rio de Janeiro; RN, Rio Grande do Norte; RO, Rondônia; RR, Roraima; RS, Rio Grande do Sul; SC, Santa Catarina; SE, Sergipe; SP, São Paulo; TO, Tocantins.

A total of 175 physicians practicing in Brazil answered the survey. The median age of the respondents was 43 years (29-80 years). Overall, 89 (50.9%) respondents were endocrinologists, 46 (26.3%) were head and neck surgeons, and 40 (22.9%) were nuclear medicine physicians. Figure 1 shows the proportional distribution of survey respondents across Brazilian states. Two-thirds of the respondents (n = 116) practiced in large cities (state capitals) with a population of ≥ 500,000 inhabitants. Most respondents (n = 109, 62.3%) practiced in both public and private institutions, while 53 (30.3%) and 13 (7.4%) worked only in private practice and public institutions, respectively. The average number of patients with thyroid cancer seen monthly by the respondents was 12 (1-120 patients).

Considering the preparation for RAI, an equal number of physicians reported either not performing a diagnostic WBS prior to RAI (n = 64, 36.5%) or doing so in selected cases (n = 64, 36.5%). A smaller group (n = 47, 26.9%) indicated that they always obtain a diagnostic WBS before RAI.

The results of the survey showed significant differences between physicians working in the public and private sectors regarding the use of rhTSH. Among those working in the public system, 92.3% (n = 12 of 13) reported rarely or never using rhTSH. In contrast, among physicians working in the private system, 73.5% (n = 119 of 162) reported using rhTSH always or in most cases, while 7.5% and 20% reported using it rarely or never, respectively, because of either lack of access to the medication or preference (p < 0.001). Additionally, 24.5% (n = 39 of 159) only use rhTSH prior to RAI and 7.5% (n = 12 of 159) only use it prior to WBS, while 67.9% (n = 108 of 159) of the physicians reported using rhTSH prior to both RAI and WBS. The remaining physicians indicated that they do not use rhTSH or do not have access to the medication. Finally, 10.4% (n = 14 of 134) of physicians only use rhTSH for patients categorized as low risk according to the ATA classification, while 40.2% (n = 54 of 134) and 49.2% (n = 66 of 134) use it for patients categorized as low/intermediate risk and low/intermediate/high risk, respectively. A total of 41 physicians did not respond to this last query.

**Figure 2:**
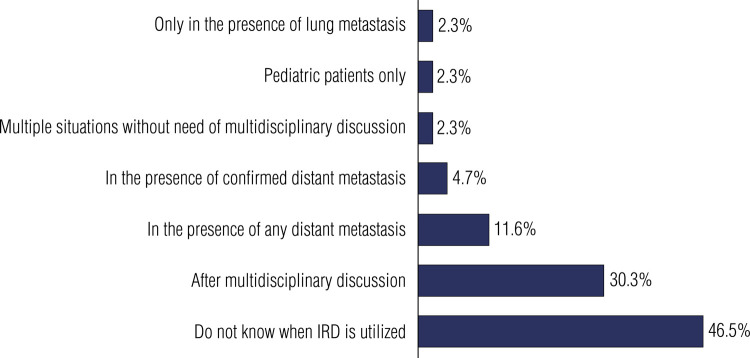
Percentage of physicians who would recommend internal radiation dosimetry protocols at different clinical indications.

Abbreviation: IRD, internal radiation dosimetry.

Protocols involving internal radiation dosimetry (IRD) are used routinely by 11.4% (n = 20 of 175) respondents, while 20.5% (n = 36 of 175) reported only using them in selected cases. Overall, 47.4% (n = 83 of 175) respondents do not use IRD protocols, and 20.5% (n = 36 of 175) indicated that they would like to use them but have no access to IRD. Of those who recommend IRD for their patients, 32.1% (n = 18 of 56) were unaware of IRD protocol specifications, 30.4% (n = 17 of 56) use whole-body IRD for maximal RAI-tolerated activity, 7.1% (n = 4 of 56) use lesion IRD to evaluate the probability of response, and 30.4% (n = 17 of 56) use both WBS and lesion IRD simultaneously. Figure 2 shows the percentages of physicians who would recommend IRD in different clinical scenarios.

When inquired about the guidelines used to determine therapeutic decisions concerning RAI, 9.7% (n = 17 of 175) of the survey respondents indicated that they prefer not following guidelines, while 75.5% (n = 132 of 175), 5.7% (n = 10 of 175), 5.7% (n = 10 of 175), and 1.7% (n = 3), respectively, reported that they base their decisions on guidelines published by the ATA, SNMMI, European Society of Nuclear Medicine, and ETA. A total of 1.7% (n = 3 of 175) reported following guidelines from other entities.

**Figure 3:**
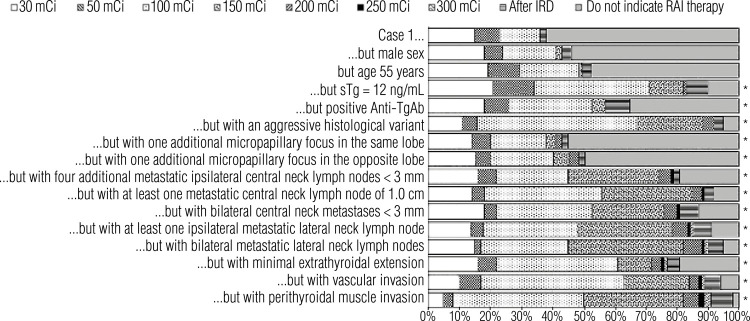
Distribution of responses regarding radioiodine treatment preferences in Case 1 and its variations.

Abbreviations: RAI, radioiodine.

Figure 3 shows the distribution of responses regarding RAI treatment preferences in Case 1 (baseline) and for the variations of this case. The baseline Case 1 presented the scenario of a 45-year-old female patient with a 2.0 cm papillary thyroid carcinoma with follicular variant histology and no other features. The variations of the case introduced different features to analyze how the respondents’ preferences in recommending RAI would change in various scenarios. When the case variations included a male (instead of a female) patient or a patient aged 55 years old, the responses did not change significantly relative to the baseline case. However, the responses changed significantly (p < 0.001) with other case variations, *e.g.*, elevated stimulated thyroglobulin level, positive antithyroglobulin antibody, aggressive histological variant, additional micropapillary foci at the same thyroid lobe, and other scenarios described in Figure 3.

**Figure 4:**
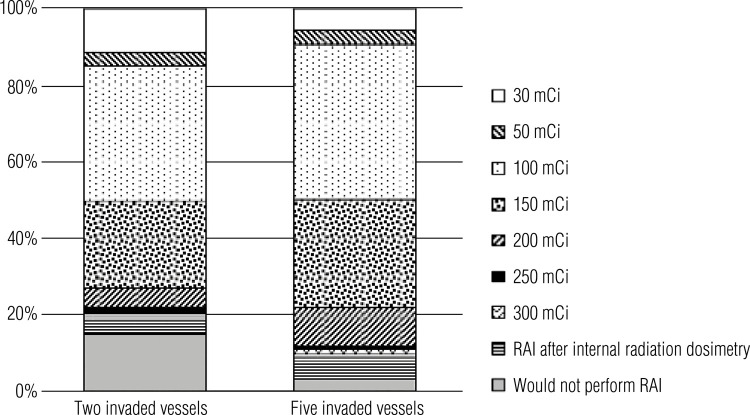
Radioiodine therapy doses indicated in Case 2 considering the scenarios of two or five invaded blood vessels.

Figure 4 shows the respondents’ RAI preferences in Case 2, which presented the case of a patient with a 3.5 cm follicular carcinoma with invasion of either two or five blood vessels. Overall, 14.9% (n = 26 of 175) of physicians would not recommend RAI with two invaded vessels, while 2.9% (n = 5 of 175) would not perform this treatment with five invaded vessels (p = 0.0017). More physicians (14.8%; n = 26 175) would indicate low-dose RAI (considered in the present study as a RAI activity of 30-50 mCi) with two invaded vessels than five invaded vessels (9.1%; n = 16 of 175). Most physicians would recommend an intermediate dose (*i.e.*, 100-150 mCi). Indeed, 35.4% (n = 62 of 175) would indicate 100 mCi with two invaded vessels and 40.6% (n = 71 of 175) would indicate the same dose with five invaded vessels, while 22.9% (n = 40 of 175) and 28.6% (n = 50 of 175) would indicate a 150 mCi dose for the cases with two and five invaded vessels, respectively. Only a few respondents would choose doses higher than 200 mCi for two invaded vessels (6.2%; n = 11 of 175) while more of them (12%; n = 21 of 175) would choose these doses if the tumor invaded five vessels.

**Figure 5:**
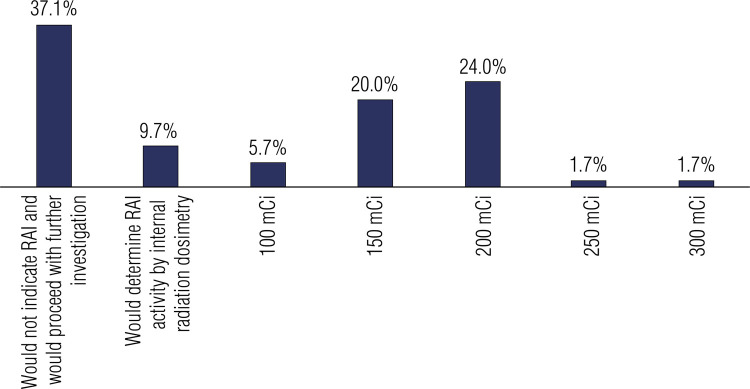
Radioiodine therapy preferences of survey respondents in Case 3.

Abbreviation: RAI, radioiodine.

Figure 5 shows the respondents’ RAI therapy preferences in Case 3. This case presented the scenario of a patient with tall-cell variant of papillary thyroid carcinoma with two metastatic level VI lymph nodes (1.2 and 0.6 cm) who had undergone therapy with a RAI activity of 100 mCi in the previous year and had a rising serum thyroglobulin of 109 ng/mL. The patient had no evidence of disease on cervical ultrasound and had scattered micronodules on chest computed tomography. The respondents were asked to indicate their recommendations for RAI therapy in the case. Most physicians (62.9%; n = 110 of 175) would recommend RAI therapy with no further investigation. The RAI dose most frequently chosen was 200 mCi (preferred by 24% of respondents, n = 42 of 175), followed by 150 mCi (20%; n = 35 of 175). Only a few respondents would recommend a lower or higher dose in this case.

**Figure 6:**
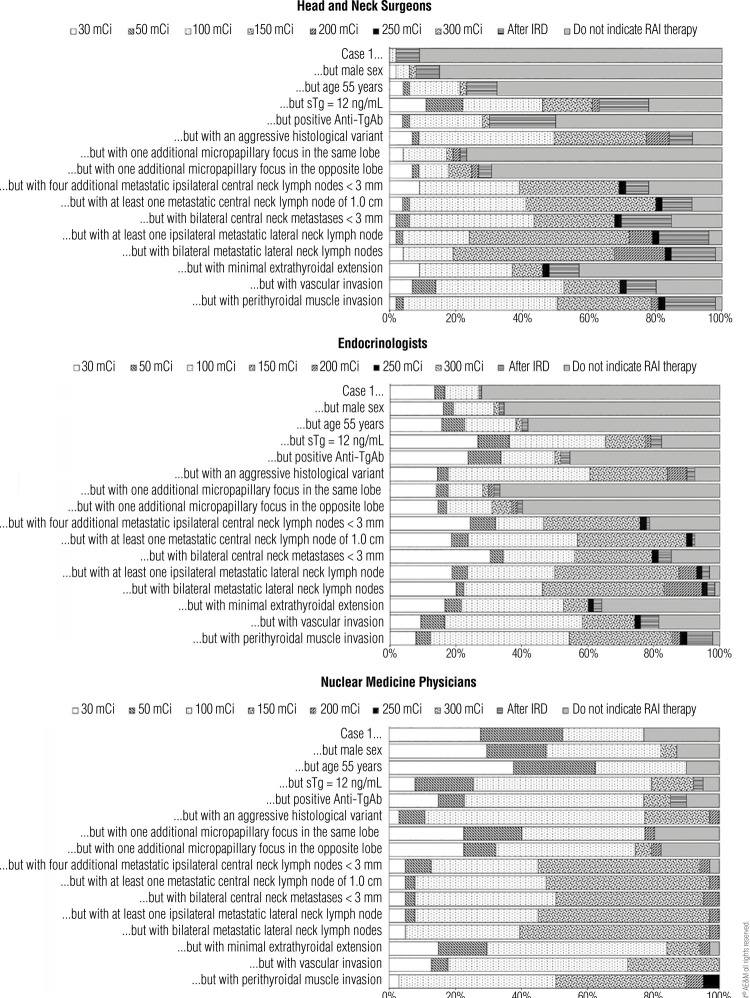
Radioiodine therapy recommendations in Case 1, as indicated by medical specialists and grouped by their respective specialties.

**Figure 7:**
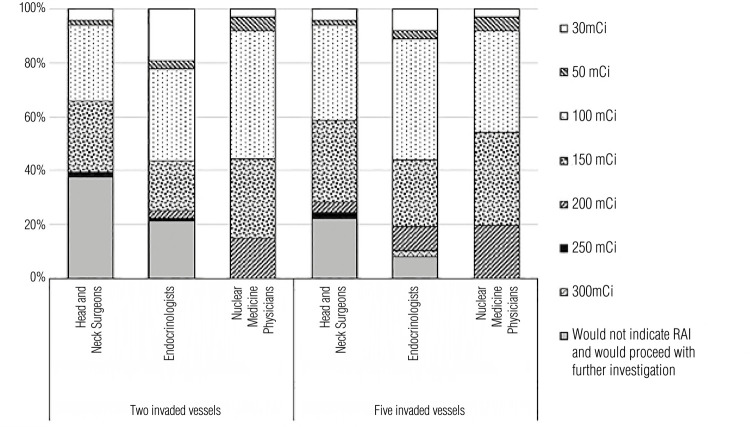
Radioiodine therapy recommendations in Case 2, as indicated by medical specialists grouped by their respective specialties.

**Figure 8:**
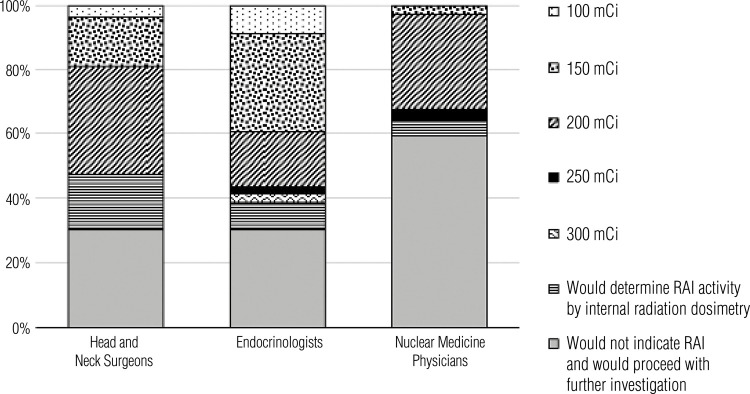
Radioiodine therapy recommendations in Case 3, as indicated by medical specialists grouped by their respective specialties.

Figures 6, 7, and 8 show the distribution of the responses to Cases 1, 2, and 3, respectively, categorized by the respondents’ medical specialties. Regarding Case 1, nuclear medicine physicians would prescribe RAI therapy more often than endocrinologists and head and neck surgeons. The RAI activity chosen was overall balanced across different specialties. In Case 2, all nuclear medicine physicians would choose RAI therapy in the cases of two or five invaded vessels. Overall, nuclear medicine physicians tended to be more aggressive in their recommendations for RAI if the tumor invaded two blood vessels, but the answers for tumors invading five vessels were similar across all specialties. None of the respondents would choose an RAI activity following a dosimetry protocol, and the most prescribed doses were 100 mCi and 150 mCi. In Case 3, most physicians indicated that they would further investigate the patient before recommending a new RAI dose, a decision that is corroborated by the ATA guidelines, which was the main guideline adopted by the survey respondents.

Table 2 compares the answers provided by physicians at each medical specialty for Case 1 and its variations. There were significant differences between specialties regarding the answers for both the baseline Case 1 and its first suggested variations (*i.e.*, male sex, age ≥ 55 years), highlighting practice differences across medical specialties. However, as the variations rendered Case 1 more aggressive, the practices across specialties became more uniform. Notably, no significant difference between specialties was observed in terms of management of the case when vascular or infiltration of perithyroidal muscles was introduced in the case.

**Table 2:** Comparison of radioiodine treatment preferences in Case 1, as indicated by medical specialists grouped by their respective specialties

	Head and neck surgeons versus Endocrinologists	Endocrinologists versus Nuclear medicine physicians	Head and neck surgeons versus Nuclear medicine physicians
Case 1	**p < 0.05**	**p < 0.05**	**p < 0.05**
Case 1 but male sex	**p < 0.05**	**p < 0.05**	**p < 0.05**
Case 1 but age 55 years	**p < 0.05**	**p < 0.05**	**p < 0.05**
Case 1 but sTg of 12 ng/mL	p < 0.05	NS	p < 0.05
Case 1 but positive anti-TgAb	p < 0.05	NS	p < 0.05
Case 1 but with an aggressive histological variant	p < 0.05	NS	p < 0.05
Case 1 but with one additional micropapillary focus in the same lobe	NS	p < 0.05	p < 0.05
Case 1 but with one additional micropapillary focus in the opposite lobe	NS	p < 0.05	p < 0.05
Case 1 but with four additional metastatic ipsilateral central neck lymph nodes < 3 mm	NS	NS	NS
Case 1 but with at least one metastatic central neck lymph node of 1.0 cm	p < 0.05	NS	NS
Case 1 but with bilateral central neck metastases < 3 mm	p < 0.05	NS	NS
Case 1 but with at least one ipsilateral metastatic lateral neck lymph node	p < 0.05	NS	p < 0.05
Case 1 but with bilateral metastatic lateral neck lymph nodes	p < 0.05	NS	p < 0.05
Case 1 but with minimal extrathyroidal extension	p < 0.05	NS	p < 0.05
Case 1 but with vascular invasion	**NS**	**NS**	**NS**
Case 1 but with perithyroidal muscle invasion	**NS**	**NS**	**NS**

Abbreviations: Anti-TgAb, antithyroglobulin antibody; NS, nonsignificant; sTg, stimulated thyroglobulin.

In Case 2, an analysis of whether physicians would alter their RAI preferences with a change in the number of invaded vessels – two *versus* five – indicated that head and neck surgeons and nuclear medicine physicians would maintain the same approaches. However, a significant shift was observed in how endocrinologists would manage their patients in these scenarios varying the number of invaded vessels (p = 0.00096). When the analysis shifted to a comparison across specialties in the same case, no differences between head and neck surgeons and endocrinologists were observed in the scenarios of two and five invaded vessels. However, differences were observed between head and neck surgeons and nuclear medicine physicians in the scenarios with two invaded vessels (p = 0.00108) and five invaded vessels (p = 0.01314), and between endocrinologists and nuclear medicine physicians in these scenarios (p < 0.0001 and p = 0.0278, respectively).

In Case 3, the differences in responses were not significant between head and neck surgeons and endocrinologists but were significant between endocrinologists and nuclear medicine physicians (p < 0.002) and head and neck surgeons and nuclear medicine physicians (p = 0.0096).

When the responses were analyzed according to the location of the respondents (capital cities *versus* non-capital cities), no significant differences were observed regarding all three cases and variations. Similarly, no significant differences in responses for all three cases were observed when the respondents were categorized according to practice in the private, public, or both sectors.

## DISCUSSION

The 2015 ATA risk of recurrence staging system is considered an important guide for the selection of patients with DTC for treatment with RAI and for the decision of the recommended RAI activity (4). The most important objective of the three-level system, originally proposed by the ATA in 2009 and later reiterated in the 2015 guidelines, was to ensure uniformity in thyroid cancer management to the greatest extent feasible. However, considerable variability in the management of these patients is still observed across countries and even in different regions within the same country. Due to that, this study aimed to understand the management practices of RAI therapy for DTC across medical specialties, health care settings (public *versus* private), and geographic regions in Brazil. This is the first study specifically analyzing the physicians’ approaches to managing DTC in Brazil.

Patients with thyroid cancer are usually cared for by specialists from a variety of clinical backgrounds. This study compared the varied practices regarding RAI therapy for DTC among endocrinologists, nuclear medicine physicians, and head and neck surgeons. The results showed that the training background makes a difference in the perception of the prescriber about recommending RAI therapy and preparing the patient for this treatment. We believe that the differences among specialists in recommending RAI treatment for DTC must be acknowledged, as highlighted by Sawka and cols. (13), as patients may receive conflicting recommendations from different physicians and potentially lose confidence in the treatment proposed by one physician.

The preparation for RAI treatment includes a low-iodine diet and achievement of an increased serum TSH level (>30 mIU/L) (4,7). The increased TSH level was initially achieved by thyroid hormone withdrawal. However, the development of rhTSH and its approval in 2007 by the US Food and Drug Administration for the treatment of patients with DTC without evidence of metastatic disease has allowed the required TSH increase to be achieved without inducing hypothyroidism with thyroid hormone withdrawal, which is associated with a poor quality of life (15). Initial studies included only patients with low-risk DTC as potential candidates for the use of rhTSH (8). However, recent studies suggest that rhTSH can also be used for patients with intermediate-risk or high-risk DTC without evidence of distant metastases (9-11). Therefore, the use of rhTSH has become a safe and effective alternative to thyroid hormone withdrawal (7). Vallejo & Muros have analyzed the cost-effectiveness of rhTSH *versus* thyroid hormone withdrawal before RAI ablation for DTC in Spanish hospitals and reported a higher incremental cost per quality-adjusted life-year (QALY) with thyroid hormone withdrawal compared with rhTSH (16). Due to the high cost of rhTSH (approximately BRL $7,000 or USD $1,400) and the fact that Brazil is a developing country, rhTSH is still unavailable in the formulary of the public health system. The analysis of rhTSH use in the public and private settings in the present study indicated that 30% of the physicians working in the public health system reported not having access to this rhTSH, with 2.3% not using rhTSH at all. In contrast, only 0.5% of the physicians working in the private setting reported not having access to rhTSH, with 1.5% reporting not using rhTSH at all. We believe that this contrast between the public and private sectors is probably due to the lack of resources in the Brazilian Unified Health System (SUS). In the present study, it was challenging to draw conclusions regarding the use of rhTSH according to health care system since a large proportion of the physicians worked in both the public and private sectors.

Regarding the use of IRD in the present study, 46.5% of physicians reported not knowing when to use it. This is highly concerning as it indicates that even physicians specializing in thyroid cancer may be unaware of beneficial techniques for treating this disease.

A low-iodine diet is part of an adequate preparation for RAI treatment. In the present study, we chose not to analyze differences among physicians regarding advice for a low-iodine diet in Brazil. The duration of a low-iodine diet for improving the expression of the Na+/ I-symporter and enhancing RAI response varies from 7 to 30 days, according to recommendations by the ATA, American Association of Clinical Endocrinologists, European Society for Medical Oncology, and British Thyroid Association (4,17-20). A Brazilian study found that a 15-day low-iodine diet is typically sufficient to deplete the iodine pool and facilitate the RAI response compared with a 30-day low-iodine diet (20). Furthermore, the assessment of the iodine pool can be effectively conducted using spot urine samples instead of 24-hour urine collections (20).

The ATA guidelines recommend that patients with a clinical suspicion of metastasis undergo pretreatment (diagnostic) WBS to determine the RAI dosing for therapy (4). A study analyzing the cost-effectiveness of this approach compared with empiric RAI dosing indicated that the approach using empiric RAI dosing is more effective in terms of costs (21). The present study showed that about one-third of the surveyed physicians reported not obtaining a diagnostic WBS prior to RAI. We did not conduct further analysis to understand how physicians’ decisions would change in cases where patients were suspected of having metastatic disease. However, we believe that differences might have emerged when considering advanced disease stages.

The best way to decide when to recommend RAI to a patient with DTC is to classify the patient as low risk, intermediate risk, or high risk. The last ATA guidelines (4) updated the risk stratification system as follows:

- **ATA low risk** (presence of all the following characteristics): no local or distant metastases, all macroscopic tumor has been resected, no tumor invasion of locoregional tissues or structures, no aggressive histology, no RAI-avid metastatic foci outside the thyroid bed on the first post-treatment WBS, if RAI is given, no vascular invasion, and clinical N0 or ≤ 5 pathologic N1 micrometastases. The patient is also considered to be low risk if the tumor is an intrathyroidal encapsulated follicular variant of papillary thyroid cancer, an intrathyroidal, well-differentiated follicular thyroid carcinoma with capsular invasion and absent or minimal (less than four foci) vascular invasion, or an intrathyroidal papillary microcarcinoma that is unifocal or multifocal, including tumors with a *BRAF* V600E mutation (if the mutation status is known).
- **ATA intermediate risk**: microscopic invasion of the tumor into the perithyroidal soft tissues, RAI-avid metastatic foci in the neck on the first post-treatment WBS, aggressive histology, papillary thyroid carcinoma with vascular invasion, clinical N1 or > 5 pathologic N1 with all involved lymph nodes < 3 cm in largest dimension, or multifocal papillary thyroid carcinoma with a *BRAF* V600E mutation (if the mutation status is known).
- **ATA high risk**: macroscopic invasion of tumor into the perithyroidal soft tissues, incomplete tumor resection, distant metastases, postoperative serum thyroglobulin suggestive of distant metastases, pathologic N1 with any metastatic lymph node ≥ 3 cm in largest dimension, and follicular thyroid cancer with extensive vascular invasion (more than four foci of vascular invasion).

For patients with low-risk DTC who have undergone total thyroidectomy, RAI is routinely not recommended, but if done, the ablation dose of 30 mCi is usually favored. If the surgery involved a partial thyroidectomy, no RAI is recommended. For patients with intermediated-risk DTC, remnant ablation doses are usually recommended, but if adjuvant therapy is required, a RAI dose up to 150 mCi could be suggested. In patients with high-risk DTC, RAI ablation should always be considered. For adjuvant therapy in these cases, a RAI dose of up to 150 mCi is usually sufficient, but for patients with known structural disease, empiric RAI doses of 100-200 mCi may be administered (100-150 mCi for patients older than 70 years). Alternatively, the RAI doses can be guided by dosimetry.

The beneficial effects of RAI therapy in patients with high-risk DTC are undisputed. However, controversy exists regarding RAI therapy in patients with intermediate-risk DTC and in some with low-risk disease. This has prompted the ETA to publish a recent consensus statement on the indications for postsurgical RAI in patients with DTC (22). One point addressed in the statement is whether patients with low-risk DTC should be treated with RAI. We understand that the main objective of ablation therapy is to facilitate follow-up studies, including measurement of serum thyroglobulin levels and RAI imaging. However, the serum thyroglobulin levels are generally undetectable after total thyroidectomy in patients on levothyroxine. In these cases, the goal of remnant ablation was already achieved with the surgery alone, thus precluding the need to perform RAI ablation. In this regard, the ETA statement is very incisive in recommending against RAI in patients with low-risk DTC who have (unifocal or multifocal) tumors < 1 cm, but RAI must be considered in the presence of abnormal neck ultrasound or elevated thyroglobulin level (22).

Patients with biochemical or structural evidence of disease after initial surgery are candidates for RAI. In contrast, patients without biochemical or imaging evidence of persistent disease may be candidates for either observation, ablation of remnants, or adjuvant therapy. In these cases, additional information should be considered. Some tests may be helpful in this assessment, including cervical ultrasound, measurement of postoperative thyroglobulin levels, and diagnostic WBS with or without SPECT or SPECT/CT (6).

An important aspect to be considered in RAI dosing decisions is the goal of the treatment, *i.e.*, ablation, adjuvant therapy, or treatment of known disease. In ablation, the objective is to treat a remnant tissue, presumably benign. Adjuvant therapy is a complementary treatment that can be used in an attempt to treat a suspected but unidentified residual disease to reduce the risk of disease recurrence or persistence. Finally, the treatment of known disease (*i.e.*, therapeutic activity) is implemented with the objective of treating known residual or recurrent disease (22).

In Case 1 (Figure 3), which included various postoperative disease statuses, the proportion of RAI indications and RAI dosing increased in all scenarios for which the risk stratification increased. These scenarios included the presence of a more aggressive histological variant, an additional focus of papillary microcarcinoma in the same or opposite lobe, four additional metastatic ipsilateral central neck lymph nodes < 3 mm, at least one metastatic central neck lymph node of 1.0 cm, bilateral central neck metastases < 3 mm, at least one ipsilateral metastatic neck lymph node, bilateral metastatic lateral neck lymph nodes, minimal extrathyroidal extension, vascular invasion, and perithyroidal muscle invasion.

Postoperative thyroglobulin levels also influenced the indication for RAI, especially in patients with intermediate-risk DTC. Undetectable thyroglobulin values indicate that the thyroidectomy was complete, the possibility of residual disease is low, and the risk of disease recurrence is very low. A stimulated thyroglobulin level < 0.5-1.0 ng/mL is reassuring and has a high probability (>98%) of identifying disease-free patients. On the other hand, a stimulated thyroglobulin level > 2.0 ng/mL suggests the presence of disease or remnant thyroid tissue and is associated with higher rates of recurrence, while increasing thyroglobulin values are also suspicious for recurrence (23-25). In one of the variations of Case 1 in the present study (Figure 3), the addition of a stimulated thyroglobulin level of 12 ng/mL unveiled differences in RAI indication among physicians from different specialties (endocrinologists, head and neck surgeons, and nuclear medicine physicians). Indeed, 21% each of head and neck surgeons and endocrinologists would still not indicate RAI even if the stimulated thyroglobulin was 12 ng/mL. On the other hand, only 5% of the nuclear medicine physicians would not indicate RAI in this scenario. Furthermore, 55% of the nuclear medicine physicians would prefer to recommend a RAI dose of 100 mCi in this situation, while only 24% of the head and neck surgeons and 36% of the endocrinologists would do the same. This shows that nuclear medicine physicians tend to be more aggressive in recommending RAI for patients with elevated stimulated thyroglobulin level. Such disparity should be emphasized in future meetings for a better unification of practices regarding RAI indications in patients with elevated stimulated thyroglobulin.

An important characteristic to be included in the pathology report of a thyroid cancer specimen is the presence of vascular invasion and the number of invaded vessels. Follicular carcinoma is a DTC of follicular thyroid cells that shows transcapsular and/or vascular invasion without nuclear features of papillary thyroid carcinoma. This type of tumor can be divided into minimally invasive (encapsulated) or angioinvasive, and the mortality rate increases according to the number of invaded blood vessels. Invasion of four or more blood vessels is associated with poorer outcomes. Follicular thyroid carcinomas with extensive vascular invasion are classified as high risk, and the indication of RAI in these cases is well established (4). In Case 2 (Table 1 and Figure 4), which described a patient with a 3.5 cm follicular carcinoma, more physicians would choose RAI and at higher doses if the patient had five invaded blood vessels compared with two. This result is aligned with the ATA recommendations for RAI treatment.

It is important to emphasize that the risk stratification of a patient with DTC varies over time. Therefore, the recurrence risk estimates assessed early in the disease should be continuously reassessed during follow-up. The categories of response to therapy include (A) excellent response (no clinical, biochemical, or structural evidence of disease), (B) biochemical incomplete response (abnormal thyroglobulin or increasing antithyroglobulin antibody levels in the absence of localizable disease), and (C) structural incomplete response (persistent or newly identified locoregional or distant metastases and indeterminate response, including nonspecific biochemical or structural findings not classified as benign or malignant) (4). Case 3 describes a patient with high-risk DTC who had a structural incomplete response 1 year after the initial treatment. Physicians from all surveyed specialties would recommend a new RAI dose for the patient, although they differed regarding the RAI dosing. As shown in Figure 5, 44% of physicians (n = 77 of 175) would recommend a RAI dose of 150-200 mCi in this case. Higher RAI doses of 250-300 mCi were rarely chosen (3.4%; n = 6 of 175), probably due to a greater risk of side effects with increasing RAI activity.

A notable finding in the present study was that the medical approaches did not differ significantly among physicians practicing in large cities compared with those practicing in smaller or countryside cities. This finding differs from that by Sawka and cols. in 2007 (13), who reported varied approaches regarding RAI indications for patients with DTC among physicians practicing across different regions in the USA and Canada. In the Sawka and cols. study, these differences were not defined by national boundaries but by well-defined differences between east–west regions and distinct cultural variations. In the present study, we found no significant differences in this regard between physicians practicing in capital *versus* non-capital cities.

We had initially expected differences in medical approach among physicians working in the public (SUS) and private health systems since the supply of service is restricted in the public system and preference is given to patients covered by private health plans (26). However, we observed no such difference. A similar result was reported by Schwengber and cols. in a study published in 2020 (14). Nearly 25% of the Brazilian population is covered by private health insurance, which led us to conclude that approximately 75% of the population relies exclusively on health care provided by the SUS. Schwengber and cols. showed that the prescription of RAI by the SUS represented 78% of all RAI prescriptions for DTC in Brazil in 2017 and 77% in 2018. Considering these percentages of RAI prescriptions, there seems to be no difference in the proportions of RAI prescriptions between the private and public sectors in Brazil. In contrast, Schwengber and cols. found that the number of RAI prescriptions per 100,000 inhabitants and the percentage of high RAI doses did not correlate with socioeconomic factors, although significant inter-state and inter-institutional variabilities in RAI use were documented (14). Overall, the present study found no significant differences between RAI practices in the public and private sectors in Brazil, but observed differing approaches in RAI indications and dosing among the specialties, mainly endocrinologists and head and neck surgeons in relation to nuclear medicine physicians.

Differences in RAI indications are so common across medical specialties that the ATA, the EANM, the SNMMI, and the ETA gathered in a meeting in Martinique in 2019 to define a set of nine principles, known as The Martinique Principles (27), which (A) describe a commitment to proactive, purposeful, and inclusive interdisciplinary cooperation; (B) define the goals of RAI therapy as remnant ablation, adjuvant treatment, or treatment of known disease; (C) describe the importance of evaluating postoperative disease status and multiple other factors beyond clinic-pathological staging in regarding RAI therapy decision making; (D) recognize that the optimal administered activity of RAI adjuvant treatment cannot be determined from the published literature; and (E) acknowledge that current definitions of RAI-refractory disease are suboptimal and do not represent definitive criteria to mandate whether or not RAI therapy should be recommended (5).

In conclusion, the indications of RAI therapy by physicians in Brazil differ across medical specialties, geographic regions, and types of health care service. The findings of our study indicate a need for a Brazilian consensus involving physicians from various specialties, similar to “The Martinique Principles.” This would help guide health care professionals in treating patients with DTC in Brazil. The creation of this guideline by one specialty alone makes no sense since the patients are seen by different physicians during treatment, and disagreements between specialties would result in patients losing confidence in their physicians. In our opinion, the creation of Brazilian principles including physicians from different specialties is the best way to unify the practices of RAI treatment for patients with DTC.
